# Correction: Hybrid chemoenzymatic heterogeneous catalyst prepared in one step from zeolite nanocrystals and enzyme–polyelectrolyte complexes

**DOI:** 10.1039/d1na90026a

**Published:** 2021-03-25

**Authors:** Margot Van der Verren, Valentin Smeets, Aurélien vander Straeten, Christine Dupont-Gillain, Damien P. Debecker

**Affiliations:** Institute of Condensed Matter and Nanosciences, UCLouvain Place Louis Pasteur 1 1348 Louvain-la-Neuve Belgium damien.debecker@uclouvain.be

## Abstract

Correction for ‘Hybrid chemoenzymatic heterogeneous catalyst prepared in one step from zeolite nanocrystals and enzyme–polyelectrolyte complexes’ by Margot Van der Verren *et al.*, *Nanoscale Adv.*, 2021, DOI: 10.1039/d0na00834f.

The authors regret that references 77–89 were incorrectly given in the original article. The correct references are listed here:

77 Y. Xue, Y. Wen, W. Huijuan, M. Liu, X. Huang, X. Ye, X. Wang and B. Li, *RSC Adv.*, 2015, **5**, 51563.

78 G. Zhang, J. Sterte and B. J. Schoeman, *Chem. Mater.*, 1997, **9**, 210–217.

79 R. Murugavel and H. W. Roesky, *Angew. Chem., Int. Ed. Engl.*, 1997, **36**, 477–479.

80 M. G. Clerici, *Kinet. Catal.*, 2015, **56**, 450–455.

81 A. B. Kayitmazer, D. Seeman, B. B. Minsky, P. L. Dubin and Y. Xu, *Soft Matter*, 2013, **9**, 2553.

82 F. Cousin, J. Gummel, D. Ung and F. Boué, *Langmuir*, 2005, **21**, 9675–9688.

83 Y. Zhang, Q. Wang and H. Hess, *ACS Catal.*, 2017, **7**, 2047–2051.

84 L. Goldstein, Y. Levin and E. Katchalski, *Biochemistry*, 1964, **3**, 1913–1919.

85 K. Kleppe, *Biochemistry*, 1966, **5**, 139–143.

86 C. L. de Vasconcelos, P. M. Bezerril, D. E. S. dos Santos, T. N. C. Dantas, M. R. Pereira and J. L. C. Fonseca, *Biomacromolecules*, 2006, **7**, 1245–1252.

87 A. Thangaraj, M. J. Eapen, S. Sivasanker and P. Ratnasamy, *Zeolites*, 1992, **12**, 943–950.

88 T. Zor and Z. Selinger, *Anal. Biochem.*, 1996, **236**, 302–308.

89 M. Bradford, *Anal. Biochem.*, 1976, **72**, 248–254.

The Royal Society of Chemistry apologises for these errors and any consequent inconvenience to authors and readers.

## Supplementary Material

